# THE QUALITY OF DAY SCALE AND THE LIVING WELL INVENTORY: TWO NEW MEASURES FOR INDIVIDUALS WITH DEMENTIA AND CAREGIVERS

**DOI:** 10.1093/geroni/igad104.3726

**Published:** 2023-12-21

**Authors:** Sheila Molony, Sam Fazio, Kimberly VanHaitsma, Rich Feinn, Sheryl Zimmerman

**Affiliations:** Quinnipiac University, Hamden, Connecticut, United States; Alzheimer’s Association, Chicago, Illinois, United States; Penn State University, State College, Pennsylvania, United States; Quinnipiac, Hamden, Connecticut, United States; University of North Carolina at Chapel Hill, Chapel Hill, North Carolina, United States

## Abstract

**Background:**

Differences in adaptive strategies used by individuals living with dementia have the potential to impact wellbeing. Two new measures were developed using human-centered design to capture daily adaptive strategies and outcomes. The Living Well Inventory for Dementia (LWI-D) and the Quality of Day Scale (QODS) capture important aspects of wellbeing that illuminate options for care and support. Purpose: To provide results from the initial evaluation of the LWI-D and the QODS for face validity, content validity and user acceptability.

**Methods:**

Acceptability and feasibility testing was conducted with a sample of 17 community-dwelling individuals with early stage dementia (MOCA scores 12-30). After measure optimization, a second pilot test was conducted with 30 dyads (persons living with dementia and family caregivers) in community and residential settings.

**Results:**

Persons with dementia can use and accurately interpret the QODS, preferring a version with graphic icons. Of the 31 items on the development version of the LWI-D, 17 items were acceptable, meaningful, and easily answered. Additional items yielded meaningful qualitative responses but needed minor revision. Some participants disliked the Likert-type response format, and new administration guidelines were used for the second pilot. Additional outcomes include convergent validity between the LWI-D and the QODS with measures of positive affect-balance, quality of life and wellbeing.

**Conclusion:**

The LWI-D and the QODS are rigorously developed promising measures that warrant further testing and may enhance the ability to a) identify strengths in living well with dementia and b) identify and test news interventions to bolster care and support.

